# Behavioral activation for smoking cessation and mood management following a cardiac event: results of a pilot randomized controlled trial

**DOI:** 10.1186/s12889-017-4250-7

**Published:** 2017-04-17

**Authors:** Andrew M. Busch, Erin M. Tooley, Shira Dunsiger, Elizabeth A. Chattillion, John Fani Srour, Sherry L. Pagoto, Christopher W. Kahler, Belinda Borrelli

**Affiliations:** 1 The Miriam Hospital, Providence, RI USA; 2 Warren Alpert Medical School of Brown University, Providence, RI USA; 3 Roger Williams University, Bristol, RI USA; 4 Brown University School of Public Health, Providence, RI USA; 5 Providence VA Medical Center, Providence, RI USA; 6 Rhode Island Hospital, Providence, RI USA; 7 University of Massachusetts Medical School, Worcester, MA USA; 8 Boston University, Henry M. Goldman School of Dental Medicine, Boston, MA USA; 9 Current correspondence address, Minneapolis Medical Research Foundation, 701 Park Avenue, S9-309, Minneapolis, MN 55415-1623 USA

**Keywords:** Smoking, Cessation, Acute coronary syndrome, Depression, Mood, Behavioral activation

## Abstract

**Background:**

Smoking cessation following hospitalization for Acute Coronary Syndrome (ACS) significantly reduces subsequent mortality. Depressed mood is a major barrier to cessation post-ACS. Although existing counseling treatments address smoking and depression independently in ACS patients, no integrated treatment addresses both. We developed an integrated treatment combining gold standard cessation counseling with behavioral activation-based mood management; Behavioral Activation Treatment for Cardiac Smokers (BAT-CS). The purpose of this pilot randomized controlled trial was to test feasibility, acceptability, and preliminary efficacy of BAT-CS vs. Standard of Care (SC).

**Methods:**

Participants were recruited during hospitalization for ACS and were randomly assigned to BAT-CS or SC. The nicotine patch was offered in both conditions. Smoking, mood, and stress outcomes were collected at end-of-treatment and 24-week follow-up.

**Results:**

Fifty-nine participants (28 BAT-CS, 31 SC) were recruited over 42 weeks, and assessment completion was above 80% in both conditions. Treatment acceptability and fidelity were high. At 24 week follow-up adjusted odds ratios favoring BAT-CS were 1.27 (95% CI: 0.41–3.93) for 7-day point prevalence abstinence and 1.27 (95% CI: 0.42–3.82) for continuous abstinence. Time to first smoking lapse was significantly longer in BAT-CS (62.4 vs. 31.8 days, *p* = 0.03). At 24-weeks, effect sizes for mood and stress outcomes ranged from η^2^_partial_ of.07–.11, with significant between treatment effects for positive affect, negative affect, and stress.

**Conclusions:**

The design of this study proved feasible and acceptable. Results provide preliminary evidence that combining behavioral activation with standard smoking cessation counseling could be efficacious for this high risk population. A larger trial with longer follow-up is warranted.

**Trial registration:**

NCT01964898. First received by clinicaltrials.gov October 15, 2013.

**Electronic supplementary material:**

The online version of this article (doi:10.1186/s12889-017-4250-7) contains supplementary material, which is available to authorized users.

## Background

Smoking cessation following an Acute Coronary Syndrome (ACS) requires increased attention. ACS patients smoke at a significantly higher rate (29–37% [1–3]) than the US population (20% [4]). Without intensive treatment, most patients return to smoking within a year following ACS [5]. Further, ACS patients are at significantly higher risk for recurrent ACS and death if they continue to smoke, with successful smoking cessation reducing mortality by 36% [6].

Depressed mood is also a major concern following ACS. Clinical and sub-clinical depression are much more common in ACS patients than the general population [7]. Symptoms of depression, even when mild, independently predict post-ACS morbidity and mortality [8]. The American Heart Association now recognizes depression as a major risk factor following ACS [7]. The two cardinal symptoms of clinical depression are negative mood and anhedonia (i.e., lack of interest/pleasure in activities, lack of positive affect). Recent evidence suggests that anhedonia symptoms may be particularly predictive of poor outcomes following ACS, with multiple findings suggesting anhedonia is more predictive than negative mood variables (e.g., [9]).

There are well established bi-directional relationships between smoking and depression/anhedonia in the general population (e.g., [10]). A recent meta analysis of 20 longitudinal studies concluded that those with depression symptoms at the time of ACS had significantly higher smoking rates post-ACS [11]. Further, smoking status partially mediates the relationship between depression and post-ACS mortality [12].

Although there are counseling treatments that address smoking and depression independently in ACS patients, to our knowledge there is no integrated treatment that addresses both. A single, integrated treatment targeting both depressed mood and smoking could be highly effective in reducing post-ACS mortality. Behavioral activation (BA) may be the ideal mood management intervention for such an integrated treatment.

BA is a counseling intervention that aims to improve mood by re-engaging patients with healthy sources of positive reinforcement in their environment. Patients and counselors work collaboratively to set individually tailored “activation goals” that are pleasurable and/or consistent with the patient's personal goals and values. Activation goals can include social outings, behaviors that advance vocation/education, exercise, hobbies, and explicit health behavior change targets (e.g., activities consistent with smoking cessation [13]).

BA efficacy has been clearly established for the treatment and prevention of depression in psychiatric [14] and medical populations (e.g., [15, 16]). BA can be delivered with fidelity by nurse-level practitioners [17], directly targets anhedonia [18], and can be effective in relatively few sessions [19]. Most importantly, recent work suggests that BA can facilitate and maintain health behavior changes among those with depressed mood (e.g., [20–22]), including a randomized trial indicating that adding BA-based mood management to standard smoking cessation treatment may be effective for smokers with elevated depression symptoms [23].

### Study aims

We systematically developed a counseling intervention that integrates standard smoking cessation counseling with BA-based mood management for post-ACS smokers; Behavioral Activation Treatment for Cardiac Smokers (BAT-CS). The present study reports the results of a pilot randomized controlled trial comparing the effects of BAT-CS to a Standard-of-Care control on smoking abstinence, as well as mood and stress. Our primary aims were to determine: a) feasibility/acceptability of the trial protocol and b) if results warrant a fully powered efficacy trial.

## Methods

### Participants

Participants were hospitalized smokers with ACS (diagnosis of unstable angina, ST and non-ST elevation MI) who were smoking before their hospitalization. Inclusion criteria were: 1) ACS diagnosis documented in the medical record, 2) smoking ≥3 cigarettes per day immediately prior to hospitalization, 3) age 18–75, 4) English fluency, 5) regular telephone access, 6) living within a 1 hour drive of the admitting hospital, and 7) willingness to “strongly consider” an attempt to quit smoking at discharge. We used a cut-off of ≥3 cigarettes per day to include regular, daily smokers who could benefit from the nicotine patch. Exclusion criteria were: 1) evidence of limited mental competency, 2) current psychosis, bipolar disorder, borderline personality disorder, or suicidality (based on chart review and self-report), 3) expectation that the participant would not live through the study period, or 4) regularly attending counseling for depression or smoking cessation and plans to continue this counseling after discharge (which would duplicate BAT-CS treatment).

### Sample size and power

This study was designed as a pilot to examine treatment feasibility/acceptability and preliminary efficacy (i.e., it was not designed to be fully powered or to establish efficacy). Specifically, with an analyzed sample size of 59 this study was underpowered (power = 47%) to detect a medium effect (i.e., OR = 2.50) on dichotomous smoking outcomes. There was greater, (although still not sufficient) power to estimate intervention effects on secondary outcomes: there was 75% power to detect a medium effect (i.e., partial eta squared = 0.13) on longitudinal continuous outcomes and 70% power to detect a medium effect (i.e., HR = 0.20) on survival analysis outcomes. Thus, primary and secondary outcome results will be presented as preliminary, and we present effect sizes in addition to significance tests. All efficacy outcomes, especially dichotomous outcomes, should be interpreted with caution.

### Procedures

All procedures were approved by The Miriam Hospital institutional review board. Participants were recruited from inpatient cardiac units at The Miriam and Rhode Island Hospitals in Providence, RI during 2013–14. Potential participants were first identified through electronic medical record reviews and medical staff referrals. Patients appearing to meet study criteria were approached by a research assistant regarding participation during their inpatient stay for ACS. Interested patients were screened for eligibility. Those that passed screening and remained willing to participate provided signed informed consent.

#### In-hospital smoking cessation counseling

All participants received one 50-min smoking cessation session in the hospital, which did not include any BA or mood management content. This session was guided by the latest version of the Treating Tobacco Use and Dependence Clinical Guidelines [24] and a National Cancer Institute self-help workbook (*Clearing the Air*) which the participant kept. During this session, counselors provided a strong and personalized recommendation to quit and discussed: 1) past attempts to quit, reframing attempts as learning opportunities, 2) risks of smoking and benefits of quitting in general and for cardiac patients, 3) personal reasons for quitting, 4) how to recognize and avoid smoking triggers, 5) how to elicit support for quitting, 6) tips for managing cravings, 7) planning for a quit date, and 8) safety/efficacy of the nicotine patch. Per treatment guidelines [24], brief motivational strategies were provided to those who remained ambivalent about quitting.

Eight weeks of the nicotine patch were offered at discharge if the patient was willing to make a quit attempt and the patient’s physician approved. While use of the nicotine patch appears to be generally safe for cardiac patients [25], there are reports of increased risk to subgroups of ACS patients [26], and clinical practice guidelines [24] recommend that the patch is “used with caution” in the 2 weeks following myocardial infarction. Thus, physician approval was obtained before patch provision. Dosing followed manufacturer recommendations (e.g., starting on 21 mg patch for those smoking >10 per day and on 14 mg for those smoking ≤10 per day). All participants who expressed interested in using the patch were cleared by their physician for patch use.

#### Randomization

We used a computer generated (using R, Cran.R-project.org) permuted block randomization procedure, with small, random sized blocks. Randomization was stratified by counselor and elevated symptoms of depression (i.e., Patient Health Questionnaire-9 (PHQ-9) ≥ 10 vs. PHQ-9 ≤ 9). The study statistician provided sequenced randomization envelopes. The randomization envelopes were opened by counselors following the completion of each in-hospital smoking cessation session. Counselors then immediately informed the participant of their treatment condition.

#### Standard-of-care (SC)

The SC group received 5 mailings of print materials at 1, 3, 6, 9 and 12 weeks post-discharge. Materials were 10 smoking cessation educational brochures produced by JourneyWorks Publishing (two sent at each time point). The 10 brochures are: 1) *Relapse Happens* 2) *Breaking Nicotine Addiction*, 3) *50 Things You Should Know About Quitting*, 4) *How Quitting Smoking Affects Your Body*, 5) *Quit Smoking Without Gaining Weight*, 6) *How Quitting Smoking Helps Your Heart*, 7) *Top Ten Steps to Quitting Smoking*, 8) *The Health Consequences of Smoking*, 9) *How to Quit When You Have Tried Before*, and 10) *Adjusting to a Smoke Free Life*. SC participants were contacted by a master’s level health educator for brief (5–10 min) “check-in” calls following each mailing. Calls focused on whether the participant received and read the written materials, general encouragement to quit smoking, and participant questions about nicotine patch use. There was no discussion of the importance of depressed mood or goal setting in the SC condition.

The treatment provided in the SC condition is above and beyond that provided by most US hospitals. However, the Joint Commission on Hospital Accreditation recently released a new performance standard [27] which encourages US hospitals to provide cessation counseling before discharge, offer cessation pharmacotherapy at discharge if medically appropriate, and assess smoking status and provide some cessation support in the weeks after discharge. SC meets all aspects of the Joint Commission standard. Our choice of a strong control group likely lowered our between group effect on smoking cessation, however it provides a more clinically and scientifically meaningful comparison.

#### Behavioral activation treatment for cardiac smokers (BAT-CS)

The BAT-CS manual was informed by the manual used in the only published RCT of BA as an aid for smoking cessation, as well as an ongoing RCT (R01DA018730) [23]. This existing manual was designed for non-hospitalized smokers and was delivered in several 90 min in-person group sessions in a community clinic. Our adaptation of this existing manual was required to account for treatment setting (e.g., initiation while hospitalized) and structure (i.e., fewer, shorter, individual counseling sessions, mostly over the phone) to better fit the needs and preferences of post-ACS patients. We assessed needs and preferences of post-ACS smokers through extensive mixed method interviews [28] and test cases. Adaptation was also guided by clear meta-analytic data on the dose of treatment needed for significant effects on smoking cessation in hospitalized cardiac patients [29, 30].

BAT-CS combines standard, guideline-driven smoking cessation counseling with BA-based mood management and goal setting techniques. BA has its underpinnings in the behavioral model of depression and purports that depression and related problem behaviors, such as smoking, can be reduced by reconnecting patients with healthy sources of positive reinforcement. BA accomplishes this through collaboratively defined between-session “activation goals” (i.e., explicitly scheduled between-session activities). In BAT-CS, activation goals had 3 targets: (1) increasing pleasant and/or meaningful activities in order to improve mood and facilitate smoking cessation, (2) increasing activities consistent with a non-smoking lifestyle (e.g., social outings in non-smoking settings with non-smoking peers), and (3) developing specific steps to prepare for and initiate a quit attempt. The BAT-CS manual is available by request from the first author [31].

All patients randomized to BAT-CS were offered a minimum of 5 post-discharge contacts at 1, 3, 6, 9, and 12 weeks. Session 2 (1 week post-discharge; 50 min) occurred in-person at a research clinic or in the participant’s home. Session 2 began with assessment of smoking status and mood (using the 2-item Patient Health Questionnaire, PHQ-2). If the patient was not smoking, the counselor reinforced their efforts, discussed successful strategies used to stay quit, and problem-solved challenges to continued abstinence. If the patient was smoking, any attempt to quit or cut down was reinforced, and relapse causes were discussed. For smoking patients interested in making another quit attempt, the counselor reviewed strategies to deal with triggers and cravings, and a new detailed plan for quitting was collaboratively developed. For smoking patients that were not interested in another quit attempt, brief motivational techniques (as specified in clinical guidelines [24]) were provided.

The counselor then reviewed the importance of addressing depressed mood after a hospitalization for ACS, presented a rationale for BA, and assessed potential targets for activation goals. Content of activation goals was determined through a personal values assessment [32] and discussion of pleasant/meaningful activities restricted due to ACS (post-ACS activity restriction has been linked to poor mood and failure to quit smoking [28]). The counselor and patient collaboratively agreed on 2–4 activation goals to improve/maintain mood and facilitate smoking cessation, to be completed before the next session, and problem-solved barriers to completion. At the end of session, the counselor provided a written list of activation goals and solutions to barriers, and offered to send the participant a between-session email or text message reminder to complete their goals.

Sessions 3–6 (3, 6, 9, and 12 weeks post-discharge; 30 min) were conducted by phone to minimize participant burden and followed the same basic format: 1) assess smoking status and depressed mood (using PHQ-2); 2) provide support for continued abstinence or smoking cessation as described in session 2 above; 3) review adherence to activation goals agreed upon in the previous session and assess reasons for failure to complete goals; 4) collaboratively choose new activation goals; and 5) problem-solve barriers to completion of new goals. Patients were offered a between-session email or text message reminder to complete goals. Session 6 (12 weeks post discharge), focused on maintenance of a high rate of behaviors that are pleasant, valued, and consistent with a non-smoking lifestyle following counseling termination.

Up to 4 additional phone booster sessions were offered (i.e. patient could choose to schedule or not) if the participant self-reported high levels of depressed mood (PHQ-2 ≥ 3) or relapse to smoking, or by participant request. The content of booster sessions was similar to follow-up calls but focused on the issue that triggered the booster session (e.g., if prompted by a high PHQ-2, its primary focus was mood management). Booster sessions were scheduled 1 week after any session where high depression or relapse was reported and lasted about 15 min.

#### Treatment fidelity

All in hospital sessions and post-randomization BAT-CS counseling sessions were conducted by the first author (licensed clinical psychologist) and second author (clinical psychology post-doctoral fellow supervised by the first author). Note that although PhDs performed all BAT-CS treatment in this pilot trial, manual content was designed for use by bachelor’s level counselors.

Attendance was tracked in BAT-CS and engagement with mailed written materials was tracked in SC. Treatment fidelity checklists were completed by counselors following the in-hospital smoking cessation session in both conditions. Following each BAT-CS session, counselors also completed treatment fidelity checklists, recorded activation goals set, and recorded participant self-report of the percent completion of each activation goal from the previous session (rated from 0 to 100% completed).

### Assessments

Participants completed the baseline assessment while hospitalized. Follow-up assessments were completed at end-of-treatment (12 weeks after hospital discharge) and 24 weeks post-discharge. Assessments were conducted by study staff blind to treatment condition.

#### Sociodemographic, medical status, and smoking history variables

At baseline (i.e., during hospital stay), participants self-reported socio-demographics and smoking history. Initial medical status and history were obtained through self-report and chart review. Nicotine dependence was measured using the Fagerstrom Test for Nicotine Dependence (FTND [33]).

#### Feasibility

Design feasibility was determined by: 1) recruitment rate, 2) percentage of those screened who qualified, 3) percentage of those qualified who chose to participate, and 4) percentage that completed follow-up assessments. Treatment feasibility was determined by number of post-discharge sessions attended in BAT-CS and number of print materials read in SC.

#### Acceptability

Treatment acceptability was assessed in both conditions at end-of-treatment using the Client Satisfaction Questionnaire (CSQ [34]), an established measure of patient satisfaction with treatment quality, quantity, and procedures. Scores range from 8 to 32 with higher scores indicating greater satisfaction.

#### Smoking outcomes

The primary smoking outcome was 7-day point prevalence abstinence (7-day PPA), defined as no smoking at all in the past 7 days, not even a puff. A breath sample was collected at follow-up assessments if the participant reported ≥7-days of abstinence. Carbon monoxide (CO) level in the breath sample verified self-reported 7-day PPA (< 10 ppm = abstinence). We made an a priori choice of CO < 10 ppm to verify abstinence as recommended by the most recent Society for Research on Nicotine and Tobacco consensus statement [35]. We are aware of some data suggesting that cutoffs as low as 3 ppm can increase certainty in determining smoking abstinence [36, 37]. However, other work indicates that for patients with Chronic Obstructive Pulmonary Disease (COPD) and other breathing disorders, these lower cutoffs may lead to a high rate of false negatives and that a cutoff of <10 ppm produces the most accurate results in this population [38]. We knew a priori from our previous pilot data that a significant proportion of our sample in this trial would have COPD, asthma, and/or shortness of breath due to heart failure. Thus, we maintained our a priori cut-off of CO < 10 ppm. Those who reported abstinence from smoking cigarettes but did not provide a breath sample or reported using other tobacco products or electronic cigarettes containing nicotine were considered smokers. We also report continuous abstinence since hospital discharge, and time to first lapse (i.e., first puff) and time to first relapse (i.e., smoking on 7 consecutive days or smoking in 2 consecutive 7 day periods), which were determined through timeline follow back interviewing methods at each assessment.

#### Mood and stress outcomes

Depression symptoms were assessed using the Patient Health Questionnaire-9 (PHQ-9) [39]. PHQ-9 scores range from 0 to 27 with higher scores indicating more depression symptoms. The standard cut-off on the PHQ-9 (i.e., ≥10 indicates likely major depression) was used to estimate the rate of major depression at baseline. The ten item Positive Affect Negative Affect Scales (PANAS) [40] assessed positive (PANAS-PA) and negative (PANAS-NA) affect at all time points. PANAS-PA scores range from 5 to 25 with higher scores indicating greater positive affect in the past week. PANAS-NA scores range from 5 to 25 with higher scores indicating greater negative affect in the past week. Perceived stress was measured using the Perceived Stress Scale (PSS [41]); scores range from 0 to 16 with higher scores indicating greater stress over the past month. We included stress as an outcome because it has been linked to poor prognosis [42].

#### Potential confounders

We tracked nicotine patch use, engagement in cardiac rehabilitation, and engagement in non-study provided counseling or medication treatment for smoking or depression during the study period. Note that we excluded based on ongoing counseling for smoking cessation or depression at baseline, but for ethical reasons, we did not *restrict* participants from seeking out additional (i.e., outside the study) counseling treatment during study participation. Thus, we have included non-study counseling for smoking cessation or depression as potential confounders.

#### BA mechanism

Behavioral activation (i.e., rate of active, pleasurable, and/or goal directed behavior) was measured using the Behavioral Activation for Depression Scale-Short Form (BADS) [43]. Scores range from 0 to 54 with higher scores indicating greater activation over the past week.

### Data analysis plan

As a preliminary step in analyzing outcomes, between group differences in baseline characteristics, medical history, and psychosocial variables were compared using *t*-tests, chi-squared tests, and Mann-Whitney U tests. Unadjusted smoking rates and unadjusted mood and stress outcomes were summarized over time by group.

For smoking outcomes we considered nicotine patch use, concurrent smoking counseling, and smoking medication use as potential confounders. For mood and stress outcomes we considered cardiac rehabilitation attendance (because cardiac rehabilitation improves mood), concurrent depression counseling, and concurrent depression medication treatment as potential confounders. Inclusion of these covariates in the final model was determined by model fit.

Using a series of longitudinal regression models implemented with generalized estimating equations (GEEs), we assessed effects of BAT-CS vs. SC on biochemically verified 7-day PPA at end-of-treatment and 24 weeks, as well as the binary indicator of continuous abstinence since hospital discharge at end-of-treatment and 24 weeks, specifying a logit link function. Models included robust standard errors to adjust for repeated measures within participant over time and adjusted for potential confounders identified a priori (final models presented below). Interest was in estimating effects (odds ratios) and corresponding 95% confidence intervals, rather than strict statistical hypothesis testing.

Secondary smoking outcomes included time to first lapse and time to first relapse. As these outcomes are time to event data, survival analysis was used to model the risk of lapsing/relapsing over time. In this case, each participant contributes two outcome variables to the model: T*_i_, time to first lapse/relapse and C_i_, censoring time. For participants who lapse/relapse before the end of treatment, T*_i_ < C_i_; for those who don’t lapse/relapse before end of treatment or who discontinue the study protocol before lapsing/relapsing, T*_i_ > C_i_. Thus, the model uses T_i_ = min (T*_i_, C_i_) as the response for each participant. Using a Cox model, we created the hazard function (which can be thought of as the number of lapses/relapses per patient-day of follow-up time) as a function of a baseline hazard rate λ_0_(t) and covariates X (t), including treatment assigned and potential confounders.

Next, using a series of longitudinal regression models implemented with GEEs [44] and identity link, we estimated the effects of treatment on mean changes from baseline in PHQ-9, PANAS-PA, PANAS-NA, PSS, and the BADS at end-of-treatment and 24 weeks. Models adjusted for confounders identified a priori and baseline value of the outcome. We also report PHQ-9 models stratified by baseline depression.

Finally, correlations between changes in BADS from baseline to end-of-treatment and 24 weeks and concurrent changes in other mood and stress outcomes were estimated to assess consistency with the BA model. We used spearman rank correlations which are less sensitive to potential outliers, which is important in small samples.

All analyses were carried out using the intent to treat (ITT) sample and run using SAS 9.3. Likelihood based approaches were used for estimation. Models made use of all available data in order to estimate effects, without directly imputing missing values.

## Results

### Sample

**Fig. 1:**
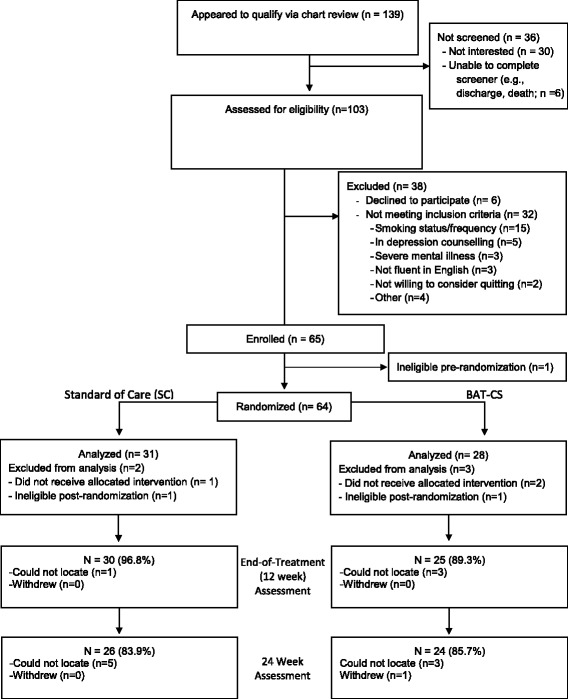
Consort diagram

One hundred thirty-nine patients were approached, 103 were screened for eligibility, 71 were eligible, and 65 consented to participate. One patient’s diagnosis was changed to a non-ACS diagnosis before randomization, thus, 64 patients were randomized. Three randomized participants did not receive the intervention (one was unexpectedly discharged to a residential rehabilitation institution with a campus smoking ban for the treatment period, two refused any treatment after discharge), and two were considered ineligible post-randomization because their final discharge diagnosis was not ACS. Thus, we report on the outcomes of 59 participants, 31 who received SC and 28 who received BAT-CS. See Fig. 1 for Consort Diagram which includes reasons for exclusion. Table 1 presents baseline data by treatment condition. No significant differences were observed between groups on any variable in Table 1.

**Table 1:** Baseline participant characteristics, mean (SD) or %

	BAT-CS (*n* = 28)	SC (*n* = 31)	Total (*n* = 59)
Demographics			
Age (years)	53.9 (11.9)	57.1 (8.3)	55.6 (10.2)
Married or living with committed partner	53.6%	58.1%	55.9%
Female	17.9%	35.5%	27.1%
Race			
Non-Hispanic Caucasian	89.3%	90.3%	89.8%
Non-Hispanic African American	3.6%	6.5%	5.1%
Hispanic Caucasian	7.1%	0%	3.4%
Multiracial	0%	3.2%	1.7%
Employed (full or part-time)	67.8%	58.1%	62.7%
Median yearly household income ^a^	$44,000	$35,500	$38,000
Some college education	46.4%	58.1%	47.5%
Medical History			
Prior ACS event	21.4%	35.5%	28.8%
Co-morbidities			
Heart Failure	10.7%	16.1%	13.6%
Diabetes	25.0%	35.5%	30.5%
COPD	25.0%	29.0%	27.1%
Previous Stroke	3.6%	3.2%	3.4%
Peripheral Artery Disease	17.8%	6.5%	11.9%
ACS Characteristics			
Type of index ACS event			
STEMI	42.9%	54.8%	49.2%
NSTEMI	46.4%	35.5%	40.7%
Unstable Angina	10.7%	9.7%	10.2%
Intervention			
Cardiac Catheterization	100%	96.8%	98.3%
≥ 1 stents placed	78.6%	87.1%	83.1%
CABG	14.3%	6.5%	10.2%
Length of Hospital Stay (days)	3.6 (4.7)	3.6 (3.0)	3.6 (3.9)
LVEF <55% ^a^	46.2%	53.3%	50.0%
Baseline Smoking			
Cigarettes/day	15.8 (9.2)	16.9 (9.5)	16.4 (9.3)
Total years smoking regularly	35.1 (13.9)	41.1 (8.8)	38.3 (11.8)
≥ Monthly Other tobacco use	3.6%	9.7%	6.8%
≥ Monthly Electronic-Cigarette use	14.3%	9.7%	11.9%
FTND	4.7 (2.4)	4.8 (2.2)	4.8 (2.3)
Baseline Mood			
Depressive Symptoms (PHQ-9)	6.8 (5.8)	7.0 (6.5)	6.9 (6.1)
PHQ ≥ 10	25.0%	29.0%	27.1%
Taking Antidepressant Medication	10.7%	9.7%	10.2%
Positive Affect (PANAS) ^b^	13.7 (4.5)	14.6 (4.6)	14.1 (19.0)
Negative Affect (PANAS) ^b^	9.7 (5.1)	7.6 (3.6)	8.6 (4.5)
Perceived Stress Scale	5.5 (3.7)	5.4 (2.8)	5.5 (3.2)
BADS	32.7 (12.7)	35.9 (10.8)	34.4 (11.7)

*Note*. *ACS* Acute Coronary Syndrome, *COPD* Chronic Obstructive Pulmonary Disease, *STEMI* ST segment elevation myocardial infarction, *NSTEMI* non-ST segment elevation myocardial infarction, *CABG* Coronary artery bypass graft surgery, *LVEF* Left ventricular ejection fraction*, FTND* Fagerstrom Test for Nicotine Dependence*, PHQ-9* Patient Health Questionnaire*-*9*, PANAS* Positive Affect Negative Affect Scales, *BADS* Behavioral Activation for Depression Scale-Short Form

^a^ *n* = 56

^b^ *n* = 58

### Feasibility and acceptability

74.1% of those approached completed screening. 91.5% of those who passed screening consented. We recruited for a total of 42 weeks, thereby recruiting 1.4 participants per week that were included in analyses. 93.2 and 84.7% of participants provided data at end-of-treatment and 24 week assessments respectively (see Fig. 1). BAT-CS participants completed an average of 4.5 (SD = 1.5) post-discharge sessions. Booster sessions were utilized by 32.1% of all BAT-CS participants. SC participants self-reported reading an average of 7.4 (SD = 2.3) of the 10 post-discharge brochures. Mean CSQ satisfaction score was 31.1 (SD = 1.3) in SC and 30.5 (SD = 2.7) in BAT-CS, a non-significant between-group difference.

### Treatment Fidelity

The treatment adherence checklist indicated that counselors provided planned in-hospital smoking cessation treatment components at a rate of 96.9% in SC and 97.3% in BAT-CS. There was no significant between-group difference in in-hospital session adherence. Counselors reported providing an average of 97.0% of planned treatment components during post-discharge BAT-CS sessions. BAT-CS participants set an average 10.6 (SD = 3.6) activation goals during the course of treatment. Average goal completion percentage was 72.7% (SD = 14.7%).

### Potential confounders

There were no significant differences between groups in: 1) use of study-provided nicotine patch (58.1% SC vs. 67.9% BAT-CS), 2) use of non-study cessation medication (25.8% SC vs. 15.4% BAT-CS), 3) engagement in concurrent smoking cessation counseling (0.0% for both conditions), 4) attendance at cardiac rehabilitation (38.7% SC vs. 38.5% BAT-CS), 5) use of antidepressants (16.1% SC vs. 15.4% BAT-CS), or 6) use of non-study depression counseling (3.2% SC vs. 7.7% BAT-CS). Although there were not between group differences, variables were still considered potential confounders of the treatment effect and were thus included as part of the final model when model fit indicated inclusion.

### Smoking outcomes

Unadjusted smoking outcomes (i.e., without covariates, assessment completers only) indicate that 48.0% of BAT-CS vs. 44.8% of SC participants achieved 7-day PPA at end-of-treatment and that 45.8% of BAT-CS vs. 42.3% of SC participants achieved 7-day PPA at 24 week follow-up. In addition, in unadjusted analyses 44.0% of BAT-CS vs. 33.3% of SC participants were continuously abstinent through end-of-treatment and 37.5% of BAT-CS vs. 34.6% of SC participants were continuously abstinent through 24 weeks.

**Fig. 2:**
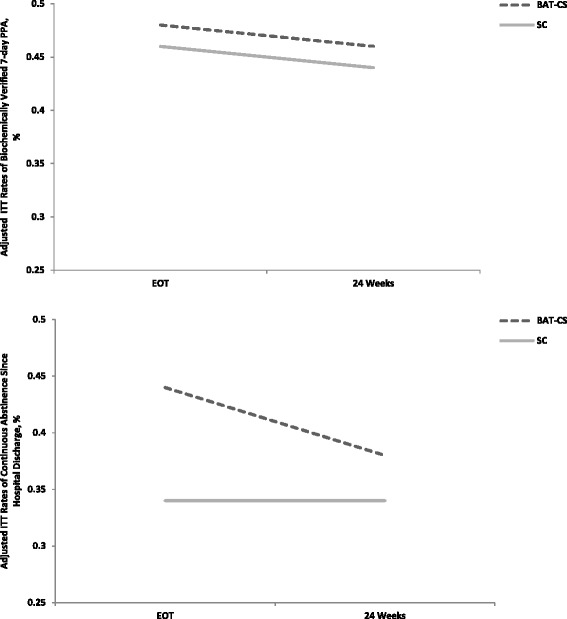
Adjusted smoking rates over time

Adjusted ITT rates of biochemically verified 7-day PPA and continuous abstinence since hospital discharge at end-of-treatment and 24 week assessments are presented in Fig. 2. There were no significant treatment effects in adjusted outcomes over time. Final models adjusted for nicotine patch use and concurrent medication treatment targeting cessation. At end-of-treatment adjusted odds ratios favoring BAT-CS were 1.12 (0.37–3.40) for 7-day PPA and 1.82 (95% CI: 0.62–5.40) for continuous abstinence. At 24 weeks adjusted odds ratios favoring BAT-CS were 1.27 (95% CI: 0.41–3.93) for 7-day PPA and 1.27 (95% CI: 0.42–3.82) for continuous abstinence.

Survival analysis results indicate that mean number of days to first lapse after discharge was significantly greater for BAT-CS vs. SC (62.4 vs. 31.8, *p* = 0.03). When modeling treatment effects on the risk of lapse after discharge, results indicate significant effects, such that for those randomized to BAT-CS, risk of lapse was 0.38 times that of those in SC (HR = 0.38, 95% CI: 0.17–0.82, *p* = 0.01). Although mean days to first relapse favored BAT-CS participants (71.2 vs. 47.4 days; *p* = 0.08), there was no significant between-group difference in the risk of relapse (HR = 0.50, 95% CI: 0.23–1.13, *p* = 0.10).

### Mood and stress outcomes

**Fig. 3:**
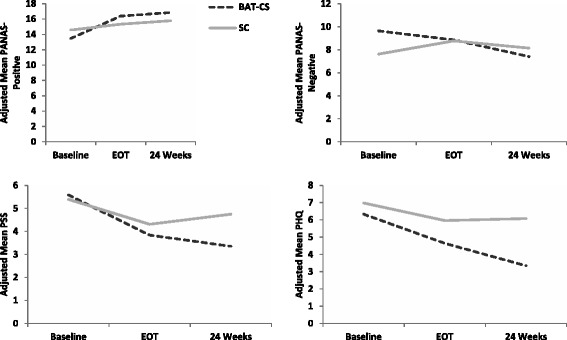
Adjusted mean mood and stress over time

Additional file 1 presents unadjusted means for all mood and stress outcomes at baseline, end-of-treatment, and 24 weeks. Fig. 3 presents adjusted means for all mood and stress outcomes at baseline, end-of-treatment, and 24 weeks. Exact adjusted means and standard errors for each time point are provided in Additional file 2. Final models for mood and stress outcomes adjusted for anti-depressant medication use and for cardiac rehabilitation attendance.

No significant differences between groups were observed for mean change from baseline to end-of-treatment for PANAS-PA (η^2^_partial_ = 0.06, *p* = 0.08), PANAS-NA (η^2^_partial_ = 0.04, *p* = 0.16), PSS (η^2^_partial_ = 0.03, *p* = 0.35), and PHQ-9 (η^2^_partial_ = 0.05, *p* = 0.72). Effect sizes were modest (η^2^_partial_ 0.03–0.06) but in the hypothesized direction favoring BAT-CS. There were significant, small to medium effects favoring BAT-CS on baseline to 24-week changes in PANAS-PA (b = 2.73, SE = 1.33, η^2^_partial_ = 0.10, *p* = .04), PANAS-NA (b = −3.02, SE = 1.34, η^2^_partial_ = 0.11, *p* = 0.02), and PSS (b = −1.80, SE = 0.82, η^2^_partial_ = 0.11, *p* = 0.03). No significant effect was observed for change in PHQ-9 from baseline to 24 weeks (η^2^_partial_ = 0.07, *p* = 0.13), but the effect was in the hypothesized direction favoring BAT-CS.

When comparing effects of treatment on changes in PHQ-9 from baseline to 24 weeks amongst those with and without likely major depression at baseline, results indicate a small, non-significant effect favoring BAT-CS in the PHQ-9 < 10 sub-sample (*n* = 43; η^2^_partial_ = 0.09, *p* = 0.06) and no effect in the PHQ-9 ≥ 10 sub-sample (*n* = 16; η^2^_partial_ = 0.01, *p* = 0.27).

### BA mechanism

Results indicate a small non-significant between-group effect favoring BAT-CS on change in BADS at end-of-treatment (η^2^_partial_ = 0.05; *p* = 0.09) and 24 weeks (η^2^_partial_ = 0.05; *p* = 0.11). Change in BADS from baseline to end-of-treatment was significantly correlated with concurrent changes in PANAS-PA (r_s_ = .27; *p* = .04), PANAS-NA (r_s_ = −.36; *p* < .01), PHQ-9 (r_s_ = −.56; *p* < .001), and PSS (r_s_ = −.51; *p* < .001). Change in BADS from baseline to 24-weeks was significantly correlated with concurrent changes in PHQ-9 (r_s_ = −.55; *p* < .001) and PSS (r_s_ = −.40; *p* < .01). Correlations of change in BADS from baseline to 24-weeks with concurrent change in PANAS-PA (r_s_ = .23; *p* = .11) and PANAS-NA (r_s_ = −.18; *p* = .22) were in the hypothesized direction, but were non-significant.

## Discussion

Results support the feasibility of this RCT protocol. Most patients were willing to be screened and most who passed the screener were willing to enroll in the study. We enrolled 1.4 participants per week of recruitment. Over 80% of participants provided primary outcome data at all assessment time points. CSQ scores indicated good treatment acceptability in both conditions with mean CSQ scores above 30 in both conditions.

All smoking outcomes were in the hypothesized direction, favoring BAT-CS efficacy. However, most were non-significant, which was expected due to limited power in this pilot study. Specifically, in fully controlled models, BAT-CS participants were 1.27 times more likely than SC participants to be both 7-day PPA abstinent and continually abstinent at 24 weeks post-discharge. There were larger effects favoring BAT-CS for continuous abstinence at end-of-treatment (OR =1.82) and risk of lapse (HR = .38, *p* = .03) and risk of relapse (HR = .50, *p* = .10) in survival analyses. When evaluating the potential clinical significance of these findings it is important to note that smoking cessation has a direct, proximal effect on mortality in this population [6]. Experts estimate that a cessation treatment that produces a 5% difference in quit rates in this population would have significant public health value [45]. In adjusted analyses, the current study indicates a 10.3 and 4.3% between treatment differences in continuous abstinence at end-of-treatment and 24-weeks respectively, suggesting potential public health significance. Further, we observed these differences despite providing a robust control condition (including 1 hour of cessation counseling, free nicotine patches, and educational materials).

As hypothesized, depressed mood and stress outcomes favored BAT-CS, with 24-week effect sizes ranging from η^2^_partial_ of .07 to.11. Differences were statistically significant for positive affect, negative affect, and stress. Change in depression symptoms at 24 weeks was not significantly different between groups, but did show a small to medium effect size favoring BAT-CS. Taken together, results indicate that BAT-CS has promise for improvement in mood and reduction in stress post-ACS. The observed effect on positive affect is particularly promising, as lower positive affect has been prospectively linked to both smoking cessation and post-ACS mortality [9, 10].

We chose to enroll patients with a range of baseline depression symptoms (i.e., from those asymptomatic to those with major depression), with the rationale that the immediate post-ACS period is a high risk time for development of depression symptoms [46], especially among smokers [47], and thus many of those without depression at baseline could benefit from BA-based prophylactic mood management. When we examined PHQ-9 changes separately for those who had likely major depression at baseline (PHQ ≥ 10 at baseline) and those that did not (PHQ < 10), the strongest effect was among those without likely major depression at baseline (η^2^_partial_ = 0.09), indicating that BAT-CS may be particularly efficacious for post-ACS depression prevention. This finding is consistent with existing trials showing that BA-based mood management can prevent depression in medical patients [16] and significantly improve mood in non-depressed individuals [18].

There was almost no 24-week between-group effect on PHQ-9 change among those with PHQ-9 ≥ 10 at baseline. This needs to be interpreted with caution given the small sample for this analysis (*n* = 16) and existing data indicating that BA-based treatment is effective for major depression in medical patients [15]. It may be the case that those with major depression would benefit from more intensive counseling than was provided in the current study.

BAT-CS had a promising effect (η^2^_partial_ = 0.05) on BA’s purported mechanisms of change measured by the BADS. While not statistically significant, this finding indicates that BA procedures provided in this study are likely affecting the targeted behavior. This result was not surprising given the high goal completion rate observed (73% in the current study; a previous successful BA for depression trial found a 58% completion rate using the same homework compliance measure [13]). We also observed correlations in the expected direction between change in BADS and change in mood and stress outcomes consistent with the core of BA theory.

This study has several limitations. First, as a small pilot study, outcome results should be interpreted as preliminary. Second, external validity was limited by the fact that counseling was conducted by Ph.D. providers (which is not the community standard of care) and by a sample that was almost 90% non-Hispanic Caucasian (which is not representative of the overall racial and ethnic diversity of the U.S.). Third, while our SC control condition exceeds what is generally available in the community, there was still significantly more contact time in BAT-CS. Finally, our longest follow-up was 24 weeks post-ACS, which limited our ability to examine the sustainability of effects.

Despite these limitations, current results are potentially impactful as they are the first data on a combined smoking cessation and mood management counseling intervention post-ACS. These results also add to the growing literature indicating that BA is an effective treatment for depressed mood in medical patients, and this is the second RCT to indicate that BA has promise for the facilitation of smoking cessation. Follow-up trials of BAT-CS should be powered to detect between group differences in mood, verified abstinence, and cardiac health at long-term follow-up and should utilize counselors similar to community providers to increase external validity. Further, future studies could explore the utility of pairing BA with counseling targeting other health behaviors known to be correlated with depression (e.g., sedentary behavior, medication adherence).

## Conclusions

This is the first study to combine smoking cessation and mood management counseling following a cardiac event. Results provide preliminary evidence that combining behavioral activation with standard smoking cessation counseling could be efficacious for this high risk population. A larger trial with longer follow-up is warranted.

## Additional files

Additional file 1: Unadjusted Mean Mood and Stress Outcomes Over Time. (DOC 30 kb)

Additional file 2: Adjusted Mean Mood and Stress Outcomes Over Time.(DOCX 14 kb)

## Abbreviations

7-day PPA: 7-day point prevalence abstinence
ACS: Acute Coronary Syndrome,
BA: Behavioral activation
BADS: Behavioral Activation for Depression Scale-Short Form
BAT-CS: Behavioral Activation Treatment for Cardiac Smokers
CABG: Coronary artery bypass graft surgery
CO: Carbon monoxide
COPD: Chronic Obstructive Pulmonary Disease
FTND: Fagerstrom Test for Nicotine Dependence
ITT: Intent to treat
LVEF: Left ventricular ejection fraction
NSTEMI: Non-ST segment elevation myocardial infarction
PANAS: Positive Affect Negative Affect Scales
PHQ-9: 9-item patient health questionnaire
SC: Standard of care control group
STEMI: ST segment elevation myocardial infarction
