# Supplementary material for: Behavioral activation for smoking cessation and mood management following a cardiac event: results of a pilot randomized controlled trial
Source: BMC Public Health. 2017 Apr 17;17:323. doi: 10.1186/s12889-017-4250-7 (PMC5392972; doi:10.1186/s12889-017-4250-7)
Supplement: Supplementary file 1 — Unadjusted Mean Mood and Stress Outcomes Over Time. (DOC 30 kb) [file 12889_2017_4250_MOESM1_ESM.doc]

**Additional File 1. Unadjusted Mean Mood and Stress Outcomes Over Time.**

|  |  | BAT-CS | SC |
| --- | --- | --- | --- |
| PANAS-Positive  Baseline  EOT  24 Weeks |  | 13.68(4.46)  16.36(2.68)  16.83(3.54) | 14.57(4.58)  15.70(4.50)  15.84(4.50) |
| PANAS-Negative  Baseline  EOT  24 Weeks |  | 9.68(5.11)  8.88(2.74)  7.30(2.55) | 7.60(3.56)  8.63(5.01)  8.20(4.04) |
| PSS  Baseline  EOT  24 Weeks |  | 5.54(3.69)  3.88(2.80)  3.30(2.93) | 5.39(2.81)  4.27(2.65)  4.75(2.77) |
| PHQ-9  Baseline  EOT  24 Weeks |  | 6.75(5.83)  4.72(4.07)  3.35(3.10) | 7.03(6.48)  5.87(5.41)  6.04(6.12) |
| BADS  Baseline  EOT  24 Weeks |  | 32.71(12.74)  38.40(9.28)  39.65(10.18) | 35.94(10.76)  37.40(11.95)  37.33(9.70) |

*Note.*  Unadjusted Mean (SD). PANAS = Positive Affect Negative Affect Scales. PSS = Perceived Stress Scale. PHQ-9 = Patient Health Questionnaire-9. BADS = Behavioral Activation for Depression Scale-Short Form. EOT = End-of-Treatment Assessment.
