# Supplementary material for: Behavioral activation for smoking cessation and mood management following a cardiac event: results of a pilot randomized controlled trial
Source: BMC Public Health. 2017 Apr 17;17:323. doi: 10.1186/s12889-017-4250-7 (PMC5392972; doi:10.1186/s12889-017-4250-7)
Supplement: Supplementary file 2 — Adjusted Mean Mood and Stress Outcomes Over Time.(DOCX 14 kb) [file 12889_2017_4250_MOESM2_ESM.docx]

**Additional File 2. Adjusted Mean Mood and Stress Outcomes Over Time**

|  | BAT-CS | SC |
| --- | --- | --- |
| PANAS-Positive  Baseline  EOT  24 Weeks | 13.47(0.68)  16.38(1.38)  16.85(1.66) | 14.56(0.47)  15.33(1.61)  15.79(1.52) |
| PANAS-Negative  Baseline  EOT  24 Weeks | 9.63(0.91)  8.86(1.03)  7.41(1.41) | 7.62(0.75)  8.77(1.03)  8.14(0.87) |
| PSS  Baseline  EOT  24 Weeks | 5.58(0.80)  3.84(1.04)  3.35(2.03) | 5.39(0.78)  4.31(1.01)  4.75(1.41) |
| PHQ-9  Baseline  EOT  24 Weeks | 6.33(2.10)  4.64(2.24)  3.34(2.62) | 6.98(1.77)  5.96(2.67)  6.08(2.35) |
| BADS  Baseline  EOT  24 Weeks | 32.77(3.45)  38.45(7.38)  39.55(6.06) | 35.97(3.25)  37.34(5.70)  37.44(4.66) |

*Note. A*djusted Mean (SE). PANAS = Positive Affect Negative Affect Scales. PSS = Perceived Stress Scale. PHQ-9 = Patient Health Questionnaire-9. BADS = Behavioral Activation for Depression Scale-Short Form. EOT = End-of-Treatment Assessment.
